# Basal ganglia calcifications (Fahr’s syndrome): related conditions and clinical features

**DOI:** 10.1007/s10072-019-03998-x

**Published:** 2019-07-02

**Authors:** Giulia Donzuso, Giovanni Mostile, Alessandra Nicoletti, Mario Zappia

**Affiliations:** Department “GF Ingrassia”, Section Neuroscience, University of Catania, Via Santa Sofia 78, 95123 Catania, Italy

**Keywords:** Fahr’s syndrome, Brain calcinosis, Basal ganglia, Hypoparathyroidism

## Abstract

Basal ganglia calcifications could be incidental findings up to 20% of asymptomatic patients undergoing CT or MRI scan. The presence of neuropsychiatric symptoms associated with bilateral basal ganglia calcifications (which could occur in other peculiar brain structures, such as dentate nuclei) identifies a clinical picture defined as Fahr’s Disease. This denomination mainly refers to idiopathic forms in which no metabolic or other underlying causes are identified. Recently, mutations in four different genes (SLC20A2, PDGFRB, PDGFB, and XPR1) were identified, together with novel mutations in the Myogenic Regulating Glycosylase gene, causing the occurrence of movement disorders, cognitive decline, and psychiatric symptoms. On the other hand, secondary forms, also identified as Fahr’s syndrome, have been associated with different conditions: endocrine abnormalities of PTH, such as hypoparathyroidism, other genetically determined conditions, brain infections, or toxic exposure. The underlying pathophysiology seems to be related to an abnormal calcium/phosphorus homeostasis and transportation and alteration of the blood-brain barrier.

## Introduction

Basal ganglia calcifications are associated with a variety of neurological and metabolic disorders, and calcifications could be also frequent incidental findings on neuroimaging of asymptomatic individuals [1]. Indeed, a certain degree of calcification of basal ganglia can be considered “physiological” with aging, over 50 years, and it could be an incidental finding in 15–20% of asymptomatic patients undergoing computed tomography (CT) scan [2–4].

Even if this condition is traditionally known as Fahr’s disease or Fahr’s syndrome, Theodore Fahr was not the first to describe brain calcifications nor contribute significantly to the understanding of this disorder. He reported a case of an 81-year-old patient with long history of dementia and post-mortem evidence of calcifications in white matter, basal ganglia, and small cerebral arteries [5], probably due to calcium-phosphorus abnormalities. Before him, in 1850, Delacour first described the presence of vascular calcifications of basal ganglia in a 56-year-old man who clinically had stiffness and weakness of lower extremities with tremor [6]. Then, in 1855, Bamberger described the histopathological entity of calcifications in the thinner cerebral vessels in a woman who had mental retardation and seizures [7].

Over the years, a variety of names have been associated with this condition; in 1974, the term “idiopathic basal ganglia calcification” (IBGC) was used for the first time [8], reporting two cases of familial idiopathic basal ganglia calcifications exhibiting features of “dystonia musculorum deformans” and used for brain calcification of unknown origin. Then, as these calcifications tend to show a predilection for the dentate nuclei and basal ganglia, a descriptive term “bilateral striopallidodentate calcinosis (BSPDC)” has been suggested [9]. In 2013, the term “primary familial brain calcification” (PFBC) has been introduced referring to a genetically confirmed neurodegenerative disorder with calcium deposits in the basal ganglia and other brain regions, in absence of a secondary cause [10]. More than 100 kindreds and sporadic cases have been reported [10], and the estimation of the minimal prevalence of PFBC from population genomic data analysis ranged from 4.5/10000 to 3.3/1000, using different level of evidence for pathogenicity [11].

Very recently, the term “primary bilateral brain calcification” has been suggested for both inherited and sporadic cases, facilitating differentiation between primary and secondary forms, and encompassing the different anatomical distribution that can be found [12]. However, considering the relative novelty of this new suggested nomenclature, in this review, we decided to maintain PFBC as the main term referring to both inherited and sporadic forms.

On the other hand, the term “syndrome” has been suggested when a secondary, and potentially treatable, cause is found [13], and it has been associated with different conditions, especially endocrine diseases. In these conditions, metabolic dysfunctions lead to an abnormal calcium/phosphorus ratio with precipitation of colloids in cerebral vessels and composition of calcified deposits [14].

In this review, we aim to clearly describe conditions characterized by basal ganglia calcification considering primary and secondary forms. A radiological description, an approach to diagnosis, and management are also discussed.

## Search strategy and selection criteria

References for this review were identified by searches of PubMed up to June 1, 2019, and references from relevant articles. The search terms *BSPDC*, *PFBC*, *IBGC*, *brain calcification*, *Fahr disease*, OR *basal ganglia calcification* were used. We carried out a second search that included all publications up to June 1, 2019, using search terms: *hypoparathyroidism*, *parathyroid*, *thyroid surgery* AND *brain calcifications* OR *basal ganglia calcifications*. There were no language restrictions and the the final reference list was generated on the basis of relevance to the topics covered in this review

## Neuroimaging features

**Fig 1:**
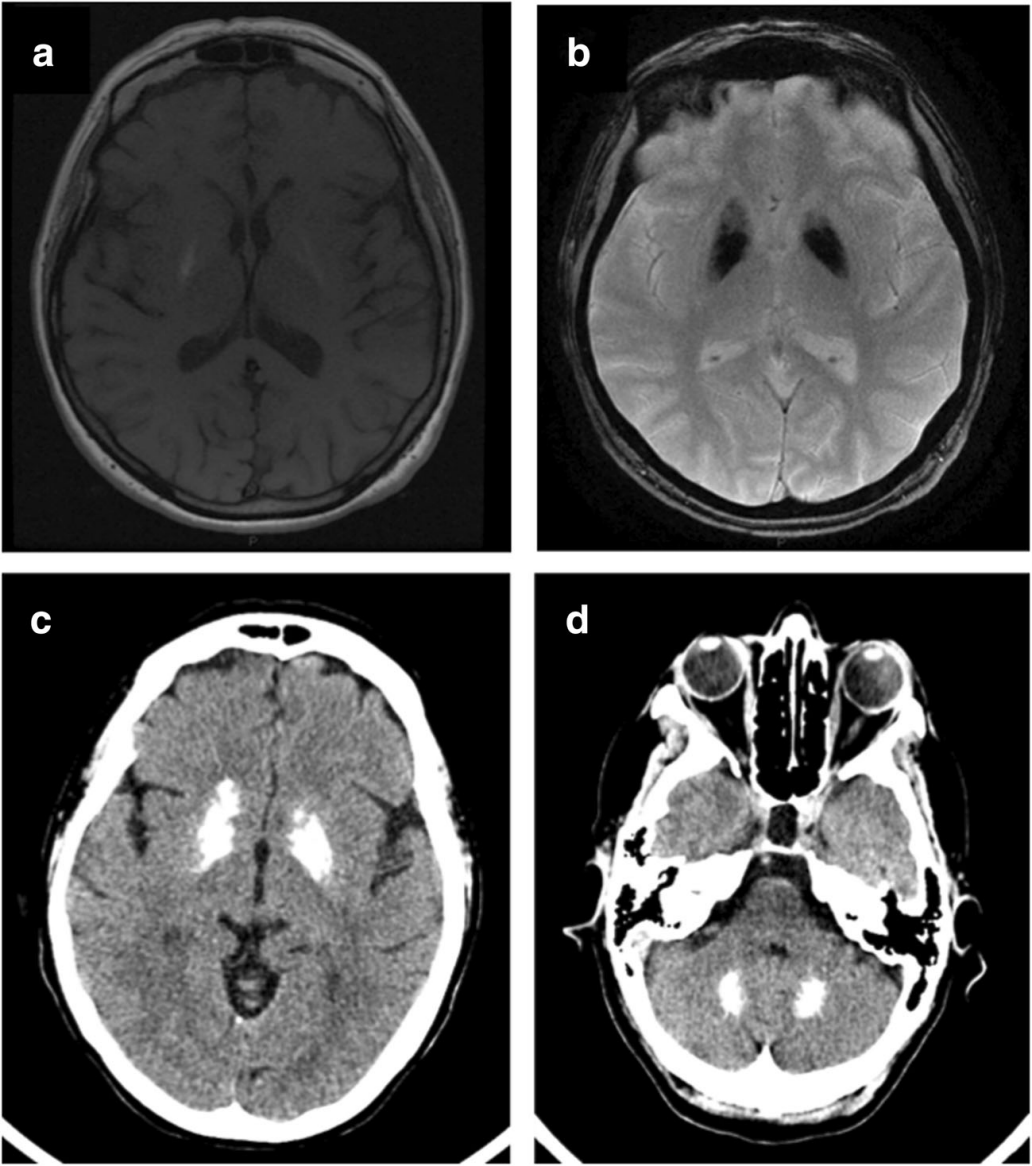
Brain MRI showing T1-w hyperintensity in basal ganglia (**a**) and severe T2*-GRE hypointensities in the same regions (**b**). Brain CT scan showing diffuse symmetric calcifications involving basal ganglia (**c**), and dentate nucleus (**d**). GRE gradient recalled echo, FLAIR fluid-attenuated inversion recovery

Since the advent of the head CT scan, the number of case reports of intracranial calcifications increased over time. CT and magnetic resonance imaging (MRI) are widely used in clinical practice to identify and quantify mineral deposition [15]. Calcified areas could be easily identified as hyperdense lesions on CT that is considered decisive for accurate diagnosis, while, studies differ in their reporting of calcium on routine MRI sequences [15, 16]. Calcium is a diamagnetic substance that may appear bright at T1-weighted imaging [17], but, at higher concentrations, the intensity of the signal in calcium at T1-weighted imaging diminishes [18]. On the other hand, calcified lesions could appear hypointense/hyperintense in T2-weighted MR images, because of the presence of other minerals in the same areas and the difference in the composition of calcium deposits, such as zinc, manganese, iron, and magnesium [15, 19, 20]. Moreover, it has been suggested that hyperintense T2-weighted lesions may be due to a progressive inflammatory process involving brain structures, which could be cause and/or consequence of calcification itself [15, 21] (Fig. 1). Recently, some authors suggested that susceptibility-weighted images (SWI), a fully velocity compensated 3D gradient-echo (GE) MR sequence with high sensitivity for blood products and iron, could definitively recognize calcifications, appearing hypointense, with higher sensitivity compared with other MRI sequences [15].

Regarding lesions’ distribution pattern and qualitative aspects, calcifications are typically bilateral and symmetrical, most frequently located in the basal ganglia, but also in dentate nuclei, thalamus, brain stem, centrum semiovale, and subcortical white matter [15]. Pathological lesions can be easily distinguished from age-related physiological calcifications of basal ganglia for their features: the latter are often small and faint, symmetrical and confined into the globus pallidus, whereas the first are more diffuse and extensive, involving also the putamen and the dentate nucleus, and usually show a coarse conglomerate pattern [22].

Cerebral scintigraphies can detect functional/metabolic abnormalities and evaluate the integrity of the nigrostriatal and striato-cortical pathways. Brain perfusion, positron emission tomography/computed tomography (PET/CT), and dopamine transporter single-photon emission computed tomography (DaT-SPECT) data showed conflicting results [9, 23–28]. In a case of Fahr’s disease with frontal lobe-type dementia and hyperkinetic-hypotonic syndrome, characterized by intermittent mild dystonic movements of hands, facial grimacing, and reduced muscular tonus of the extremities, ^18^F-fluorodeoxyglucose (^18^F-FDG) PET/CT showed reduced glucose uptake not only in putamen and globus pallidus but also in temporal and parietal cortices, bilaterally [23]. The authors suggested that the reduced glucose cortical uptake could be secondary to the impaired circuits’ function involving the basal ganglia [23]. These findings were consistently confirmed by subsequent studies [24, 25, 27, 28] showing a perfusion deficit in basal ganglia regions detected by Tc-99m HMPAO brain perfusion [28] and by ^18^F-FDG PET [29] suggesting that calcifications could play a critical role in the pathogenesis of neuronal degeneration. Le Ber and coll [25] showed a significantly reduced ^18^F-FDG uptake in striatal and cortical areas, including the precuneus, posterior cingulate, and superior temporal gyri, in patients with familial calcifications and cognitive and psychiatric symptoms.

Similarly, the evaluation of the dopaminergic system and striatal uptake showed heterogeneous results. In two patients with PFBC, ^18^F-fluoro-L-dopa uptake did not reveal any difference between patients and control subjects suggesting no evidence of dysfunction of the nigrostriatal dopaminergic pathway [9]. Saito and coll [26] used PET with [^11^C]-labeled 2*β*-carbomethoxy-3*β*-(4-flurophenyl)-tropane ([^11^C]CFT), a ligand for the striatal pre-synaptic DA transporter, and [^11^C]-labeled raclopride ([^11^C]RAC), which binds DA D2 receptors and reflects postsynaptic DA function, in a case of PFBC with psychiatric symptoms and drug-induced parkinsonism. PET scanning showed a severe decline in pre- and postsynaptic dopaminergic function in bilateral striatum, suggesting the disruption of cortico-subcortical circuits [26]. Regarding DaT-SPECT with ^123^I-ioflupane and a possible nigrostriatal involvement, there were also conflicting results [27–29]. Two studies [27, 28] found a reduction in DaT binding in striatum regions in patients with basal ganglia calcifications presenting with parkinsonism and cognitive impairment; on the other hand, it has been reported normal striatal uptake, despite the presence of diffuse calcifications of basal ganglia documented bilaterally by a CT scan in patients with psychiatric symptoms and tremor [29] and with parkinsonism and cognitive impairment [30].

These results suggest that the different patterns of brain perfusion and dopaminergic function could be related to brain plasticity and neuronal adaptation to calcifications, leading to different structural and functional neuroimaging features.

## Neuropathology

Recent neuropathological data suggest that PFBC is a disease of the cerebral microvessels, involving first vascular smooth muscles cells and pericytes. Moreover, very recently, hypodermal microvessel calcifications within and around the pericytes in the basal lamina in skin biopsies from patients with a genetic form of familial calcifications have been shown [31]. Histopathological studies showed that calcium is the major element present and it accounts for the radiological appearance of the disease, together with the involvement of several minerals like iron, magnesium, aluminum, and zinc [1, 32]. Macropathologic examination showed gray discoloration and gritty consistency of the posterior periventricular region, globus pallidus, putamen, and anterior thalamus and mild atrophy of the caudate nucleus, as well as in the cerebral cortex [33]. Reactive astrocytes and microglia accumulated around the calcified deposits, indicating a mild ongoing inflammatory process [34]. Calcifications were also observed in the tunica media of medium- and small-caliber arteries, arterioles, and capillaries leading to the obstruction of the lumen [35]. In addition, analysis of the blood-brain barrier showed that extravasation and perivascular deposition of fibrinogen was focally associated with areas of calcification [34]. Similar findings had been shown also in patients with brain calcifications due to pseudopseudohypoparathyroidism [36] and longstanding hypoparathyroidism [37], suggesting a common neuropathological pathway involving pericytes and endothelial environment both in primary and secondary forms.

## Classification

In 2005, Manyam proposed a classification based on the anatomical sites where calcification occurred, identifying bilateral striopallidodentate calcinosis, striopallido (“basal ganglia”) calcinosis, and dentate (cerebellar) calcinosis [1]. Otherwise, in order to identify possible biochemical abnormalities or reversible causes that result in deposition of calcium, a classification based on etiology has been proposed, i.e., primary/genetic forms of basal ganglia calcinosis, which includes autosomal dominant, familial and sporadic forms, and secondary forms associated with different diseases [1] (Table 1).

**Table 1:** Etiological classification of basal ganglia calcifications

Primary forms	
Gene	Chromosome position
SLC20A2	8p11.21
PDGFRB	5q32
PDGFB	22q13.1
XPR1	1q25.3
MYORG	9p13.3
Secondary forms	
	Calcium/phosphorus abnormalities
	- Idiopathic or secondary hypoparathyroidism
	Infections (brucellosis, AIDS, toxoplasmosis, TORCH complex)
	Toxic exposure (lead, carbon monoxide)
	Disimmunopathies (SLE)
Other conditions	
	Pseudohypoparathyroidism
	Cockayne syndrome I and II
	Aicardi-Goutières syndrome
	Mitochondrial diseases (MELAS, MERRF)
	Coat’s syndrome
	Neuroferritinopathy, NBIA

*SLC20A2*, solute carrier family 20 member 2; *PDGFB*, platelet-derived growth factor beta; *PDGFRB*, platelet-derived growth factor receptor beta; *XPR1*, Xenotropic and Polytropic Retrovirus Receptor 1; *MYORG*, Myogenic Regulating Glycosilase; *MELAS*, mitochondrial encephalopathy, lactic acidosis, and stroke-like episodes; *MERRF*, myoclonic epilepsy with ragged red fibers; *NBIA*, neurodegeneration with brain iron accumulation; *SLE*, systemic lupus erythematosus

### Primary forms

Primary form refers to PFBC. PFBC manifests as autosomal dominant, familial, and sporadic forms; in these cases, there is the absence of metabolic or other secondary causes. Sporadic forms might be due either to de novo mutations or to a mutation transmitted by an undiagnosed, asymptomatic parent [1, 2, 38]. Genetic analyses of large families affected by autosomal dominant brain calcifications identified 2 loci named idiopathic basal ganglia calcification (IBGC) 1, located in chromosome 14q48 [39] and IBGC 2, in chromosome 2q37 [40]. In the following years, mutations in three other genes have been found to cause the disease: solute carrier family 20 member 2 (SLC20A2) within the IBGC 3 locus, platelet-derived growth factor receptor beta (PDGFRB), and platelet-derived growth factor subunit beta (PDGFB) subsequently designated as IBGC 4 and IBGC 5, respectively [41]. Additionally, mutations in Xenotropic and Polytropic Retrovirus Receptor 1 (XPR1) were identified and designated as IBGC 6 [42, 43]. Recently, sequencing analysis revealed a linkage between IBGC 1 and IBGC 3 [44] and identified multiexonic SLC20A2 deletions in the IBGC2 kindred [45], so that IBGC 1 and IBGC 2, identified as false loci, now all map to the SLC20A2 gene (IBGC 3). SLC20A2, located at 8p11.21, encodes the inorganic phosphate transporter PiT-2, a transmembrane protein associated with phosphate homeostasis in various tissues, including the brain, and its mutations result in a reduction of phosphate transport [38]. Mutations in SLC20A2 gene are responsible for most cases identified so far and over 40 pathogenic variants have been reported in patients with PFBC [46, 47]. Microarray analysis provided evidence that the neuroanatomical pattern of expression for SLC20A2 is highest in the regions most commonly affected in PFBC, showing that globus pallidus had the highest expression among basal ganglia, followed by thalamus and cerebellum [48].

PDGFRB (5q32) and PDGFB (22q13.1) are involved in pericytes recruitment, blood-brain barrier regulation, and angiogenesis [49, 50]. XPR1 (1q25.3) is a gene encoding a retroviral receptor with phosphate export function and involved in phosphate homeostasis [42]. Very recently, mutations in the Myogenic Regulating Glycosylase (MYORG) gene were identified as a novel genetic cause for autosomal-recessive PFBC in Chinese patients [51, 52]. MYORG gene encodes a glycosyl hydrolase involved in myogenesis and expressed throughout the brain, particularly in the cerebellum, but its role in the pathogenesis of PFBC is yet unknown [53].

A systematic review from 2012 (when genes for PFBC were discovered) to November 2016 collected 137 cases, of which 34 were familial, and showed that SLC20A2 was the most common gene involved in 75 out of 137 cases (54.7%), followed by PDGFB (43 cases—31.4%) and PDGFRB (13 cases—9.5%). XPR1 gene mutations were present in 6 cases (4.3%) [54]. Over the years, several case reports described mutation variants, with peculiar clinical presentation, in SLC20A2 [55–58], PDGFB [59, 60], PDGFRB [61, 62], and XPR1 [63, 64].

The clinical phenotype encompasses neurologic and psychiatric features. Neuropsychiatric symptoms could be present in the form of schizophrenia like psychosis, depression, and irritability, and patients with extensive calcification seem to exhibit a higher frequency of psychiatric disorders than patients with limited involvement of brain structures [10]. Neurologic disturbances are heterogeneous; movement disorders are more commonly described, but seizures, headache, and cerebellar symptoms may occur. Neuropsychiatric features are also variable and include cognitive impairment, mood disorders, psychotic symptoms, and obsessive-compulsive symptoms [38]. Table 2 shows the types and frequencies of symptoms in patients with genetically proven PFBC observed in three recent studies including sporadic and familial cases [54, 65, 66]. In particular, genotype-phenotype analysis showed parkinsonism seemed to be more common in SCL20A2, occurring up to 70% of patients, with the involvement of basal ganglia, thalamus, and dentate nucleus [65, 66]. Analysis of cases reported with PDGFB mutations had hyperkinetic movements up to 25% of cases, cerebellar symptoms and headache, but also psychiatric manifestations, with calcifications of basal ganglia and cerebellum, while PDGFRB abnormalities were associated with depression, cognitive impairment, and headache. XPR1 mutations showed cognitive dysfunction (66.7%) with hyperkinetic movements and dysarthria and cortical calcifications [54]. Moreover, patients with an early onset presented most frequently with psychiatric or cognitive symptoms, while older patients exhibited mostly movement disorders [67]. Considering only movement disorders, parkinsonism was found up to 85%, chorea in 19%, tremor in 8%, dystonia in 8–19%, athetosis in 5%, and orofacial dyskinesia in 3% of patients [66–68]. Furthermore, affected members of the same family with a novel SCL20A2 mutation presented with similar brain calcification distribution but different cognitive and behavioral signs [69]. Later, Ramos and coll [70], by screening the well-known PFBC genes in four cohorts from America and Europe, found known and novel mutations of SLC20A2, PDGFB, PDGFRB, and XPR1, confirming the higher prevalence of SLC20A2 as the major causative gene (up to 13%) and, consistent with previous series, the most frequent symptoms were parkinsonism (52.3% of symptomatic individuals), cognitive impairment (40.9%), and psychiatric signs (38.6%) [70].

**Table 2:** Clinical features of patients with genetically confirmed primary familial brain calcification

No. of cases	57 cases (22 families) [51]			167 cases (25 families) [55]			137 cases (34 families) [47]			
	SLC20A2	PDGFB	PDGFRB	SLC20A2	PDGFB	PDGFRB	SLC20A2	PDGFB	PDGFRB	XPR1
	24	19	14	119	32	16	75	43	13	6
Symptomatic (% within the mutation)	17 (70.8)	11 (57.8)	5 (35.7)	71 (59.7)	23 (71.9)	7 (46.7)	57 (76)	32 (74.4)	6 (46.1)	6 (100)
Seizures^a^	0 (0)	1 (9.9)	0 (0)	3 (2.5)	0 (0)	0 (0)	5 (8.7)	2 (6.2)	0 (0)	0 (0)
Cognitive impairment^a^	11 (64.7)	6 (54.5)	2 (40)	18 (25.3)	5 (7)	0 (0)	6 (10.5)	6 (18.7)	1 (16.7)	4 (66.6)
Parkinsonism^a^	12 (70.6)	3 (27.3)	2 (40)	16 (22.5)	3 (13)	2 (28.6)	16 (28)	3 (9.3)	1 (16.7)	3 8(0)
Tremor^a^	6 (35.3)	2 (18.2)	1 (20)	NA	NA	NA	14 (24.5)	8 (25)	1 (16.7)	1 (16.7)
Dystonia^a^	6 (35.3)	2 (18.2)	1 (20)	22 (30.9)	7 (30.4)	0 (0)	10 (17.5)	4 (12.5)	0 (0)	0 (0)
Ataxia/cerebellar symptoms^a^	1 (5.9)	2 (18.2)	1 (20)	7 (9.8)	4 (17.4)	2 (28.6)	6 (10.5)	6 (18.7)	0 (0)	1 (16.7)
Chorea^a^	1 (5.9)	1 (9.1)	1 (20)	NA	NA	NA	9 (15.7)	6 (18.7)	1 (16.7)	1 (16.7)
Dysarthria^a^	5 (29.4)	3 (27.2)	1 (20)	NA	NA	NA	5 (8.7)	1 (3.1)	0 (0)	2 (33.3)
Headache^b^	6 (25)	5 (26.3)	3 (21.4)	16 (13.4)	14 (43.8)	3 (20.0)	3 (4)	14 (32.5)	3 (23.1)	0 (0)
Psychiatric symptoms^a^	13 (76.4)	8 (872.7)	4 (80)	19 (26.7)	5 (7)	0 (0)	9 (15.7)	13 (40)	3 (50)	2 (33.3)

Data shown as *n* (% within the mutation); ^a^among symptomatic patients; ^b^among all patients. *NA*, not available; *SLC20A2*, solute carrier family 20 member 2; *PDGFB*, platelet-derived growth factor beta; *PDGFRB*, platelet-derived growth factor receptor beta; *XPR1*, Xenotropic and Polytropic Retrovirus Receptor 1

Very recently, Guo and coll [64] described the mutational spectrum of the first four discovered causative genes in a series of PFBC in Chinese patients, including 35 families and 191 sporadic individuals. They found different prevalence of mutations and symptoms than those of previous studies [54, 65, 66], underlining the heterogeneity of genotype-phenotype correlation. SLC20A2 mutations were found in 14.2% of all patients, while mutations in the other three genes were very rare, accounting for 0.9% of all patients, respectively. Among symptomatic patients (44.8%), the most common symptoms were chronic headache (23.3%), movement disorders (20%), and vertigo (16.7%); moreover, total calcifications score was related to clinical symptoms and was higher in patients with SLC20A2 mutations [64].

Differently from the other identified mutations, MYORG mutation carriers showed a very homogenous clinical presentation [71]. Dysarthria was present in 100% of symptomatic patients, together with the development of other features including akinetic-hypertonic syndrome (100%), cerebellar syndrome (66.6%), gait disorders, and pyramidal signs (86.6%). Moreover, they showed a distinct radiological pattern including cerebellar atrophy and severe extensive calcifications involving the pons [71].

This wide spectrum of clinical presentation, even in the same pedigree in which individuals shared the same causing mutation, could be due to the different pathogenicity caused by different types of mutations, penetrance and expressivity, and “resilience” brain ability of individuals [72], and may depend on location of calcium deposits [54] but no obvious association between the site of calcification on CT and the type of clinical sign was observed [66]. Moreover, although Tadic and coll (2015) showed that penetrance of the imaging phenotype was 100%, interestingly, almost a quarter of cases included in the analysis were asymptomatic, suggesting reduced penetrance of the mutations and/or incomplete reporting and/or unrecognized clinical symptoms and signs in carriers of mutations [66]. The spectrum of clinical symptoms of PFBC is further complicated by the presence of known mutations in a second gene that may contribute to or be entirely responsible for the disease manifestation [73], such as SLC20A2 and the neighboring THAP1 gene in a large dystonia family [74] or changes in the epilepsy-linked SCN2A gene in combination with SLC20A2 variants in PFBC patients with refractory seizures [75].

These data also support the evidence that genetic forms of brain calcification could be underestimated suggesting the importance of future studies in asymptomatic patients carrying genetic mutations.

### Secondary forms

Secondary forms of brain calcifications have been associated with different conditions. In these cases, the term “Fahr’s syndrome” has been proposed by some authors, to describe the heterogeneity of neuropsychiatric and neuroradiological features associated to the underlying causes [1, 54]. The clinical history, examination, and laboratory findings are crucial for differential diagnosis. It is possible to identify secondary forms associated with endocrine disorders, especially hypoparathyroidism, leading to calcium/phosphorus abnormalities or other secondary conditions, such as infectious or toxic agents, when a documented exposition has been shown.

#### Calcium/phosphorus abnormalities in hypoparathyroidism

Calcium/phosphorus (Ca/P) ratio abnormalities, particularly due to parathyroid hormone (PTH) disorders, appear to be the most common definable etiology for calcification of white matter and bilateral subcortical nuclei [68]. The principal function of PTH is maintenance of calcium plasmatic levels, withdrawing the calcium from bone tissue, reabsorbing it from the glomerular filtrate, and indirectly increasing its intestinal absorption by stimulating active vitamin D (calcitriol) production [76]. Additionally, the PTH promotes an increase in urinary excretion of phosphorus and bicarbonate [77].

The most common causes leading to calcium homeostasis disorder include idiopathic or secondary hypoparathyroidism. Idiopathic hypoparathyroidism is an uncommon condition with a prevalence of 37 per 100.000 [78], characterized by the absence, fatty replacement, or atrophy of the parathyroid glands [13]. In a study of 147 patients with idiopathic hypoparathyroidism, 107 patients (73.8%) showed basal ganglia calcifications on CT scan and its occurrence and progression were associated with low Ca/P ratio [79]. On the other hand, long-term hyperphosphatemia could lead to down regulation of phosphate transporter in basal ganglia, thus determining colloid precipitation in cerebral blood vessels and brain calcification [14].

Secondary hypoparathyroidism is a relatively frequent complication of total or subtotal thyroidectomy with an incidence ranging from 6.9 to 46% for transient hypoparathyroidism, and from 0.9 to 1.6% for permanent hypoparathyroidism [76]. In an analysis of 170 consecutive patients undergoing primary total thyroidectomy, 41 patients (24.1%) developed transient hypoparathyroidism and 2 patients (1.2%) developed permanent hypoparathyroidism [80]. Epidemiological studies estimate the incidence of hypoparathyroidism related to neck surgery in the USA, which is reported in 7–36% of surgeries, of which 38% are due to total thyroidectomy, 21% parathyroidectomy, 9% partial thyroidectomy, and 5% other neck surgeries [78]. Consequently, the most common clinical scenario underlying basal ganglia and dentate nuclei calcifications is secondary hypoparathyroidism due to thyroid surgery with destruction or vascular compromise of parathyroid tissue, with an abnormal Ca/P ratio, leading to hyperphosphatemia and hypocalcaemia [81]. Symptoms due to hypocalcaemia include paresthesia, cramps, spasm of carpal and pedal muscles, seizures, neuromuscular irritability, and ECG abnormalities such as prolonged QT interval and neurological signs [30, 82–92]. CT scan shows bilateral symmetrical calcifications involving basal ganglia, thalamus, corona radiata, and subcortical white matter and cerebellum [30, 82–92] and a variety of neurological signs and symptoms were described, such as seizures, loss of consciousness, falls, gait disturbances and postural instability, cognitive decline, parkinsonism, and rest tremor [30, 82–92]. Although calcifications of basal ganglia were present in all cases, clinical features were different and heterogenic suggesting that there is no clear correlation between brain areas involved and clinical signs.

#### Infections

Brain infections, such as brucellosis, acquired immune deficiency syndrome (AIDS), toxoplasmosis, but also intrauterine and perinatal congenital infections (toxoplasmosis, rubella, cytomegalovirus, or herpes simplex virus—TORCH complex) could determine brain calcifications [10]. Brucellosis is a zoonosis that affects animals as the primary host (e.g., sheep, goats) and humans as the secondary host. Neurobrucellosis occurs in 5–10% of cases of brucellosis and affects the central or peripheral nervous system. This may lead to a variety of clinical manifestations and imaging abnormalities that mimic other neurologic diseases [93]. Brain calcifications are rare, involving basal ganglia, dentate nucleus, and white matter, and the detection in individuals residing in endemic areas should raise the possibility of brucellar infection involving the central nervous system [94].

Immunodeficiency caused by human immunodeficiency virus type 1 (HIV) determines a broad spectrum of clinical and pathological features. Pathologically, the terminal HIV-infected patient usually shows severe depletion of lymphoid tissue and opportunistic infections. Calcification is one of the most common pathological findings and involves blood vessels of multiple organs, including the brain [95]. Up to 33% of HIV-infected children show bilateral and symmetrical basal ganglia calcifications, involving putamen and globus pallidus and are usually not seen before 10 months of age [96]. In a retrospective autopsy study, 85 HIV-infected adult brains were examined and calcifications were observed in four cases (5%, calcific vasculopathy in three cases and parenchymal calcification in one case) [97].

Acute infection with Toxoplasma gondii usually is asymptomatic in children and adults, but serious clinical disease could result from a congenital infection. Infants with congenital toxoplasmosis are asymptomatic in about 70 to 90% of the cases, but they could develop sequelae during childhood or early adult life. A prospective study including 43 infants and children, with a confirmed diagnosis of congenital toxoplasmosis, showed diffuse intraparenchymatous calcifications in 28 children (65%) and the presence of calcifications in the meninges and areas of the caudate nucleus indicates a poor prognosis [98].

#### Toxic exposure

Although the pathophysiological mechanism of brain toxicity related to chronic lead exposure is poorly understood, it has been suggested that a prolonged exposition could cause intracranial calcifications in adults [99]. Tonge and coll [100] showed microscopically a significant correlation between cerebellar calcification and increased lead levels in bones in 10 to 15% of autopsies performed between 1951 and 1976. Neuroimaging findings include multifocal curvilinear calcifications in the subcortical white matter of both cerebral and cerebellar hemispheres and the involvement of basal ganglia, bilaterally [101]. A post-mortem neuropathological study on a 72-year-old man who was diagnosed with lead poisoning showed macroscopic and microscopic calcifications in the cerebellum, cerebral cortex, gray–white junction, white matter, basal ganglia, and thalamic regions. The pattern of calcification included deposits consisting of small calcospherites in the walls of the capillaries and in the tunica media of small- to medium-caliber vessels [102]. Basal ganglia calcifications involving globus pallidus may occur also in carbon monoxide poisoning [103].

#### Disimmunopathies

Systemic lupus erythematosus (SLE) is a chronic autoimmune multifactorial disease characterized by multisystemic involvement and diverse clinical features [104]. Involvement of the nervous system with cognitive and/or psychiatric, central, and peripheral nervous is referred to as neurolupus [105] and has been reported to be associated with brain calcification up to 30% of patients [106]. Brain CT scan showed the presence of diffuse calcifications over the basal ganglia, centrum semiovale, and cerebellum, and the involvement of the cerebral cortex [107–109].

### Other conditions

Basal ganglia calcifications related to other genetically determined conditions occur mainly with other systemic signs or symptoms.

#### Pseudohypoparathyroidism

Brain calcifications can also occur in genetically determined forms of resistance to PTH defined as pseudohypoparathyroidism, in which clinical and laboratorial hypoparathyroidism findings (hypocalcemia, hyperphosphatemia) are associated with normal or high plasmatic levels of PTH that is normally produced [77]. This condition had been linked with mutation in GNAS (guanine nucleotide binding protein G alpha stimulating) and STX 16 (syntaxin), causing the occurrence of seizures, movement disorders with or without cognitive impairment, or psychiatric symptoms together with other clinical features such as short stature and skeletal abnormalities [110].

#### Cockayne syndromes

Cockayne syndrome (CS) type I (moderate CS) is characterized by basal ganglia calcifications, developmental abnormalities in the first two years, progressive impairment of vision, hearing, and central and peripheral nervous system functions leading to severe disability. CS type II (severe CS or early-onset CS) overlaps with cerebro-oculo-facioskeletal syndrome (COFS) or Pena-Shokeir syndrome type I and it is characterized by growth failure at birth, with little or no postnatal neurologic development. Congenital cataracts or other structural anomalies of the eye may be present. CS type III (mild CS or late-onset CS) is characterized by essentially normal growth and cognitive development or by late onset [111].

Cockayne syndromes can result from mutations in either the excision repair cross-complementation group 6 (ERCC6) gene or the ERCC8 gene involved in repairing damaged DNA, but early reports found no obvious genotype-phenotype correlations for mutations in either *ERCC8* or *ERCC6*, suggesting that the clinical variability within the CS spectrum may not be accounted for gene mutation alone [112]. CS is classified among the childhood leukodystrophies, and brain imaging findings are cardinal features suggesting the diagnosis of CS [113]. The main clinical findings are increased tone/spasticity, hyper- or hyporeflexia, abnormal gait or inability to walk, ataxia, tremor, seizures and behavioral abnormalities, and hearing and visual loss [111, 112, 114]. In patients with classic CS, symmetric, attenuated, and homogeneous putaminal calcifications are usually predominant, while in patients with severe CS II and COFS, diffuse cortical calcifications at the depths of the sulci are associated with the putaminal calcifications [113].

#### Aicardi-Goutières syndrome

Aicardi-Goutières syndrome (AGS) is a familial progressive early-onset encephalopathy characterized by physical and mental abnormalities, seizures, calcification of basal ganglia (particularly putamen, pallidus and thalamus), leukodystrophy, cerebral atrophy, and progressive microcephaly [115]. Crow and coll have identified mutations in 4 genes encoding the exonuclease TREX1 (*AGS1*) on chromosome 3 and all 3 subunits of the hetero-trimeric ribonuclease H2 (RNaseH2) endonuclease complex (*AGS2* on chromosome 13, *AGS3* on chromosome 11, *AGS4* on chromosome 19) as responsible for the AGS phenotype [116]. These genes provide transcript for nucleases and absent or impaired enzyme function may result in the accumulation of unneeded DNA and RNA in cells [117]. In AGS, calcifications are more frequently small and punctuate in the basal ganglia and in the deep and periventricular white matter [118].

#### Mitochondrial diseases

Mitochondrial disorders could be characterized by abnormalities in calcium metabolism and high level of serum lactic acid. Mitochondrial encephalopathy, lactic acidosis, and stroke-like episodes (MELAS) and myoclonic epilepsy with ragged red fibers (MERRF) have been associated with calcification of the basal ganglia occurring up to 13% of cases [119]. MELAS is due an A-G point mutation at nucleotide 3243 (A3243G) in the tRNA^Leu^ gene of mtDNA (MTTL) [120]. A family with features of both MERRF and MELAS (MERRF/MELAS overlap syndrome) and a mutation in the gene MTTS1 (transfer RNA, mitochondrial, serine 1) whose members showed bilateral calcification of basal ganglia and cerebral atrophy has been described [121].

#### Coat’s disease

*Coat’s disease* is an eye disorder characterized by abnormal development of the retinal blood vessels. The underlying cause is not known but some cases may be due to somatic mutations in the *NDP* gene (Norrie Disease Pseudoglioma, encoding for a protein called norrin, participating in chemical signaling of cells) [122]. Goutières et al. described two children with bilateral Coats’ disease associated with cerebral calcifications in the basal ganglia and deep white matter, asymptomatic at the time of their discovery [123].

#### Neurodegenerative conditions

Some neurodegenerative conditions such as neurodegeneration with brain iron accumulation (NBIA) and neuroferritinopathies could present basal ganglia calcification due to excess iron storage, neuroinflammation, or cystic degeneration, mainly in putamen or globus pallidus [13]. Recently, two cases of genetically confirmed pantothenate kinase-associated neurodegeneration (PKAN) with globus pallidus calcification have been described [124, 125]. Basal ganglia calcifications have also been described in two patients with beta propeller-associated neurodegeneration (BPAN), a recently characterized type of NBIA caused by mutations in the tryptophan-aspartic acid (W-D) dipeptide repeats domain 45 (WDR45) gene encoding for β propeller protein, presenting with progressive dystonia and dementia and autistic regression and seizures, respectively. Neuroimaging findings showed the presence of bilateral dense calcification of globus pallidus revealed by CT scan together with symmetric MRI T2 hypointensity of bilateral globi pallidi and substantia nigra [126, 127]. PKAN and other NBIA syndromes should be considered in the differential diagnoses of basal ganglia calcification especially when calcification is limited to the globus pallidus with early onset of neuropsychiatric features characterized by slowly progressive extrapyramidal signs and psychiatric symptoms [124, 125].

Recently, Shi and coll [128] recently described a novel mutation in Ras-related protein 39B (RAB39B) as a potential cause of X-linked juvenile parkinsonism with very early onset presenting with bilateral pallidum calcifications. RAB39B, a member of the RAS oncogene family, consists of 2 exons on the chromosome Xq28 and plays an essential role in neuronal maintenance and survival [128].

## Diagnostic approach

The detection of bilateral basal ganglia calcifications on CT/MRI investigations, together with a progressive neurologic dysfunctions, which generally includes movement disorders and/or neuropsychiatric manifestations, should start a diagnostic workup based on key points, orienting in the differential diagnosis between primary or secondary forms [1].

Since idiopathic or secondary hypoparathyroidism are the most common causes of basal ganglia calcification in adulthood, disorders of Ca/P metabolism and parathyroid abnormalities should be excluded [68, 81]. Specific blood test is crucial to exclude poisoning-related brain calcifications, infections, or autoimmune conditions [129]. If secondary causes are excluded and the family history is suggestive of autosomal dominant inheritance, molecular genetic testing should be considered [13]. SLC20A2 is the most common gene involved followed by PDGFB and PDGFRB [54]. Finally, age-related and asymptomatic physiological basal ganglia calcifications have been detected up to 20% of routine CT scan and the radiological pattern could distinguish them from pathological calcifications [3]. Usually, physiological calcifications involve the globus pallidus and are bilateral and faint; other regions that may be involved include the pineal gland, falx, and choroid plexus [129].

## Therapy

Management strategies and treatment mainly focus on symptomatic relief and are strictly related to the clinical features. Since selective removal of deposited calcium from the brain without effecting calcium from bone and other tissues appears to be an impossible task [1], pharmacological treatment should be used to improve neurological and/or psychiatric symptoms and to try to remove underlying cause [10]. The presence of an abnormal Ca/P metabolism or parathyroid dysfunction should be corrected with intravenous calcium gluconate or long-term oral therapy with calcium and calcitriol, with the improvement of extrapyramidal signs and chronic paresthesia or seizures in most cases [82, 86–88]. Additionally, appropriate antiepileptic drugs for seizures should be used [89]. Antiparkinsonian drugs could be useful for patients with parkinsonism, even if pharmacological response could be minimal [83, 88, 130]. Patients who develop psychiatric features should be treated with mood stabilizer or antipsychotic drugs. Neuroleptic medication should be used cautiously, since they may exacerbate extrapyramidal symptoms, while a poor response to neuroleptics was obtained in a family with brain calcifications and psychotic manifestations [10]. Loeb et al. used disodium etidronate in one patient, determining symptomatic benefit without reduction in calcification [130]. Recently, a case series of 7 patients treated with alendronate showed good tolerance and evidence of improvements in symptoms (tremor and headache) and stability by some patients, suggesting the effectiveness of alendronate therapy in basal ganglia calcification [131].

## Conclusions

Basal ganglia calcification is a rare neurodegenerative disorder occurring as primary/idiopathic disease or as secondary manifestation of a condition, leading to an abnormal deposition of calcium in peculiar brain structures. This condition is associated with a variety of neuropsychiatric signs and symptoms and could be unnoticed for years. Recently, genetic mutations have been identified helping in distinguish between idiopathic and secondary forms, associated with different conditions. Clinically, parkinsonism or other movement disorders appear to be the most common presentation, together with seizures and cognitive decline. CT scan is considered the gold standard for diagnosis identifying calcifications as hyperdense lesions typically bilateral and symmetrical, most frequently involving basal ganglia, but also dentate nuclei, thalamus, and subcortical white matter. The anatomical pattern of calcifications does not seem to be directly correlated to the clinical phenotype. Further studies are needed in order to clarify pathological mechanisms of calcium deposition and identify specific therapeutic strategies.
