# Correction to: Basal ganglia calcifications (Fahr’s syndrome): related conditions and clinical features

**DOI:** 10.1007/s10072-019-04037-5

**Published:** 2019-08-23

**Authors:** Giulia Donzuso, Giovanni Mostile, Alessandra Nicoletti, Mario Zappia

**Affiliations:** Department “GF Ingrassia”, Section Neuroscience, University of Catania, Via Santa Sofia 78, 95123 Catania, Italy

**Correction to: Neurological Sciences (2019)**

10.1007/s10072-019-03998-x

The article “*Basal ganglia calcifications (Fahr’s syndrome): related conditions and clinical features, written by Giulia Donzuso, Giovanni Mostile, Alessandra Nicoletti, and Mario Zappia*”, was originally published electronically on the publisher’s internet portal (currently SpringerLink) on 3 July 2019 without open access. With the author(s)’ decision to opt for Open Choice the copyright of the article changed on August 2019 to *©The Author(s) 2019* and the article is forthwith distributed under the terms of the Creative Commons Attribution 4.0 International License (http://creativecommons.org/licenses/by/4.0/), which permits use, duplication, adaptation, distribution and reproduction in any medium or format, as long as you give appropriate credit to the original author(s) and the source, provide a link to the Creative Commons license and indicate if changes were made.

The original article has been corrected.

**Open Access** This article is distributed under the terms of the Creative Commons At tribution 4.0 International License (http://creativecommons.org/licenses/by/4.0/), which permits unrestricted use, distribution, and reproduction in any medium, provided you give appropriate credit to the original author(s) and the source, provide a link to the Creative Commons license, and indicate if changes were made.

